# Considerations for the translation of nutrient recommendations as dietary plans for infants, children, and adolescents as reported in Italian Guidelines for healthy eating

**DOI:** 10.3389/fnut.2022.935963

**Published:** 2022-08-25

**Authors:** Laura Rossi, Deborah Martone, Raffaela Piccinelli, Pasquale Buonocore, Andrea Ghiselli, the Working Group on Pediatric Nutrition of Italian Dietary Guidelines, Margherita Caroli

**Affiliations:** Research Centre for Food and Nutrition, Council for Agricultural Research and Economics (CREA), Rome, Italy

**Keywords:** nutrient reference intake, pediatric age group, dietary plans, dietary guidelines, Italy balanced dietary plans for pediatric age

## Abstract

Healthy eating in childhood and adolescence is important for proper growth and development and to establish a healthy foundation for future dietary habits. This paper aims to describe the elaboration of dietary plans for pediatric ages highlighting critical points related to the application of the Italian nutrient recommendations. National databases on food composition data were used to compile the dietary plans. Starting from the definition of serving size for adults, the suggested portion size for pediatric ages was shaped to produce appropriate dietary plans according to the different classes of age. Items from “cereals and derivatives,” “milk,” “fruit,” “vegetables,” “olive oil,” and “water” groups were included daily. Pizza was included once a week. Sweets and snacks were gradually introduced, once a week in infants and up to five times a week in adolescents. Legumes were provided three times a week, as a source of vegetable and sustainable proteins. The main critical aspect of the dietary plans was related to the monotony, especially for snacks and breakfasts. This work demonstrated the limits of the translation of Italian nutrient recommendations into dietary plans for pediatric age groups. Keeping the protein intake in the recommended range of 8–12% of daily energy resulted in inadequate provision of calcium and iron. Other critical points were the reference values for body weight and physical activity. The proposed dietary plans are adequate with regard to food composition, the intake of energy and several nutrients, and in terms of food selection. However, their implementations require action, information, and advocacy.

## Introduction

Dietary guidelines (DGs) have the purpose of making consumers aware of the principles of healthy diets and providing direction for programs and policies to ensure food security for all. Children of all ages have unique, age-specific nutritional needs and challenges (1). Nutrition is important at every age; although healthy eating in childhood and adolescence is more important for proper growth and development and to prevent various health conditions (2). Proper nutrition for children can also help establish a foundation for future healthy eating habits and appropriate knowledge on dietary principles that could be maintained throughout life (3, 4).

A structured process for developing DGs was first introduced in 1998 by the World Health Organization (WHO) and the Food and Agriculture Organization (FAO) (5). This process was more recently and concisely described by the European Food Safety Authority (EFSA) (6). The translation of nutrient population goals into food-based dietary guidelines is a complex process that must combine scientific findings from nutrition research with practical components related to food behaviors and preferences (7). For that, the development of DGs requires the synthesis and analysis of a wide range of evidence and contextual information; the process also requires the involvement of multiple stakeholders from relevant sectors and could have both technical and political components (8, 9).

A summary of the recommendations as well as a description of the development process was published for, among others, the United States (10, 11), Canada (12), Netherlands (13), Thailand (14), and China (15). Moreover, the comparative approach of different countries in developing the DGs was reported by Bechthold et al. (16), who evaluated DGs in Europe, or Herforth et al. (17) who provided a global review of DGs and assessed the alignment of guidelines with key elements of a healthy diet for the general adult population as articulated by the WHO (18). These key elements include the consumption of fruits and vegetables (at least 400 g or 5 portions/day), legumes, nuts, and whole grains, a moderate intake of fat and small amounts of added sugars, and salt. Forty percent of countries have separate messages or guidelines for specific subpopulations, which include infants younger than 2 years, school-aged children, adolescents, pregnant and lactating women, the elderly, and others. Among the 92 countries with DGs at the FAO repository, half provide separate guidance for age and/or physiological groups. Only some of the guidance for specific groups is developed and disseminated as part of the national DGs. In other cases, and particularly in high-income countries, government guidance for specific groups (most commonly, infants, young children, and pregnant and lactating women) may be developed through parallel processes (1).

Another comparative work was carried out by Montagnese et al. (19) who, after an analysis of the European food-based dietary guidelines, concluded that more emphasis should be given to some subgroups of the total population who represent a clear prevention target such as children and adolescents. Schwartz et al. (20) evaluated to what extent international and national guidelines cover infants’ and children’s feeding habits. They concluded that guidelines, in general, cover most topics, but some of the national guidelines are incomplete and it could be an opportunity to give more practical tips to parents, especially to help them establish responsive feeding behavior. Innovative aspects of the dietary guidelines development were reported by both Schwingshackl et al. (21), who analyzed the inclusion of the plant-based food in the DGs, and by da Silva Oliveira and Silva-Amparo (22), who provided a comparative analysis between the DGs for the Brazilian Population published in 2006 and 2014. In this framework, it is interesting to consider the work carried out by Kastorini et al. (23), which, starting from Greek DGs addressed to the general population, developed specific dietary guidelines and recommendations on physical activity for promoting healthy habits in infants, children, and adolescents as well as proposing menus based on traditional Greek diet.

Italy revised and published the national DGs in 2018 (24). The Italian Dietary Guidelines (IDGs) have been conceived and developed to include all age groups from infants to the elderly, including physiological conditions such as pregnancy and lactation, promoting the principle of a healthy diet across all ages of life. In this comprehensive approach, it was decided to have a specific focus on infants, children, and adolescents, considering that the inclusion of dietary plans, intake frequencies, and suggested portion sizes (SPSs) would represent an added value for a policy document such as the Dietary Guidelines (25).

Infants, children, and adolescents were considered under the definition of pediatric ages that Italy has ratified and made executive by law (26) based on the UN Convention on the Rights of the Child (27), which claimed that “a child is any individual under the age of 18 years unless he or she has reached maturity earlier under the applicable legislation.” The definition of pediatric age is not univocal and depends on the legislative or scientific references of each country. A study by the European Academy of Pediatrics (28), providing a comparative evaluation of the health systems of the 29 European countries, pointed out that the majority of them (15) defined the pediatric age as up to 18 years, while in the rest of the European countries, the maximum pediatric age ranged from 14 to 19 years. These differences are related to the tendency to consider the anatomical, physiological, and clinical characteristics of the child rather than only the chronological age (29). Otherwise, the definition of pediatric age could be related to the possibility of benefiting from extended health assistance. In this sense, the position of the American Academy of Pediatrics (30) is to recommend, in consideration of the cost of the healthcare, continuing the assistance up to the age of 21 years.

In Italy, pediatric nutrition actions are mainly concentrated on infants younger than 2 years. The aspects of infant feeding largely addressed by national documents are the promotion of exclusive breastfeeding up to 6 months of age (31) and complementary feeding (32). The manuals approaching childhood nutrition (33, 34) mainly address qualitative recommendations related to nutrient intakes and general aspects of children feeding practices. concerning pediatric nutrition, an important role was carried out by the school meal system that has a long tradition in Italy. Italian National guidelines for school meals prepared by the Ministry of Health were first released in 2010 (35) and then recently updated (36). These documents, however, were conceived as informative and not normative with different degrees of application at the regional level according to local autonomy (37–39). The documents concerning these school meal programs were developed at regional levels and provided menus covering lunch and, in some cases, the morning break snack, for a variable period of 4–9 weeks in winter and 4–9 weeks in spring. Food quantities and frequencies were reported for different classes of age covering the kindergarten and primary school (3–10 years). These documents were used as the starting point of this work, especially for the development of portion sizes. The limits of what is currently available in Italy are evident, with the absence of dietary plans covering the whole day for all classes of age as well as the absence of uniformity in the definition of portion size and consumption frequencies for pediatric ages.

To address these limitations, the reasons for the work reported in this paper has as its main objective the description of the process of elaboration of dietary plans for pediatric age as part of IDGs, highlighting the critical points related to the application of Italian Dietary Reference Values (DRVs) of the Nutrient and Energy Reference Intake Levels for the Italian population (40) in the class of age 1–17 years, as well as the difficulty of combining nutritional recommendations and acceptance in these ages. We considered it relevant to show the process of developing dietary plans and portion size recommendations for this class of age in Italy because, before the publication of the fourth revision of the IDGs (24), an official national document providing this information did not exist. The articulation of the process, the methodology, the criticisms, and the assumptions that were undertaken for the development of dietary plans for the pediatric age range are reported and discussed in this paper which is intended for a scientific audience, different from the target audience of the Guidelines which are practical and applicative. The specific purpose of this paper is to provide tables reporting suggested consumption frequencies and SPSs for each food typology and different classes of age (infants, children, and adolescents) as well as to show the congruencies of the proposed plans with the Italian DRVs. With this paper, we also intended to stimulate a revision of the Italian DRVs in which recommendations that are proposed nutrient by nutrient would not sufficiently take into account the combinations of different recommendations once applied in dietary plans covering the whole day or the whole week.

This paper contributes to the research discussion related to the limitations and obstacles of the practical application of nutrient dietary recommendations into daily dietary plans for pediatric ages following the IDGs. The research questions underlying this work are (i) to what extent is it possible to translate evidence from nutrient-based research into the dietary guideline for pediatric ages? (ii) To what extent it is possible to adapt pediatric nutrient recommendations into nutritionally adequate diets for children? and (iii) once the methodological criticisms are overcome, what is the level of acceptance of the proposed dietary plans by the target age groups?

## Materials and methods

### Target ages, nutritional recommendations, food frequencies, and suggested portion size

The dietary plans proposed in this paper cover the pediatric age group (1–17 years) which are referred to as infants, children, and adolescents. In the initial compilation of the dietary plans, a 3-year age breakdown was used in accordance with the age divisions in the Italian DRVs (40). However, during the progression of the work, it became evident the need to split the “1–3 years” age group into two distinct groups given the great differences in Italian DRVs of such a heterogeneous age group. Thus, six classes of ages were considered: 12–23 months and 24–47 months (infants); 4–6 years and 7–10 years (children); and 11–14 years and 15–17 years (adolescents). The Italian DRVs were used as a reference for energy, macro, and micro-nutrient recommendations for each age class.

Energy requirements for the pediatric age include the total energy expenditure (TEE) and the energy deposited in the growing tissues. In the calculation of the energy (En) requirement values (kcal/day), the median values of the physical activity level (PAL) were considered. The final numbers provided are referred to as the average for the age group and among the sexes.

For proteins, the Italian DRVs were identified with the factorial method, taking into account the needs for growth. Proteins were expressed as g/kg body weight/day [population reference intake (PRI)] and as % of daily energy intake [reference intake range for macronutrients (RI)].

Total fat was expressed as RI (% En) and as suggested dietary target (SDT) (% En) when it was possible to set population prevention goals for specific age classes and/or typology of fats.

For total carbohydrates, it was taken into account the RI (% En) and for total sugars (sum of sugars naturally present in foods and added sugars) the SDT (% En) was considered. For dietary fiber, the recommendation is expressed as Adequate Intake (AI–g/day).

For minerals (calcium, phosphorus, iron, magnesium, and zinc) and vitamins (A, B_6_, B_12_, C, D, E, and Total Folate), DRVs are expressed as PRI (mg/day or μg/day), except for vitamin E expressed as AI.

Starting from the definition of serving size for adults, the SPSs for infants, children, and adolescents were shaped to produce dietary plans adequate to the different classes of age and adapted to the nutritional needs of the pediatric age. The dietary plans resulting from this exercise were reported in the IDGs (24) to provide examples of weekly sets of food choices aimed to inform parents about the provision of children’s healthy and balanced diet.

### Food groups and food composition

According to the Italian Federation of Nutrition Societies (41), a “food group” is defined as a category into which different foods may be placed according to their similar origin of production, similar nutritional properties, similar marketing characteristics, or all three. Each food group provides a range of nutrients, and all have a role in helping the body function. In the preparation of the present dietary plans, it was considered that every day it is necessary to include foods from all groups to cover the nutritional needs of the target groups (42). The food grouping system used for dietary plan preparation is reported in Supplementary Table 1 of the Supplementary Material in which the included subgroups are shown. In summary, the food grouping included three levels of food aggregation: seven main food groups, 12 food groups, and 22 food subgroups. The main food groups consisted of (i) cereals (and derivatives) and tubers, (ii) fruit and vegetables, (iii) meat, fish, eggs, and legumes, (iv) milk (and derivatives), (v) seasoning fats, (vi) nuts and seeds, and (vii) water. Groups and subgroups were derived from the main groups. Among the food subgroups, a further selection was carried out to include food particularly adequate for pediatric age, excluding, among others, bread substitutes, added fats different from extra-virgin olive oil, etc. The dietary plans for infants, children, and adolescents presented in this paper were mainly based on “basic foods,” different from the so-called “discretionary foods” or “non-necessary foods,” a concept introduced in the last revision of IDGs (25). The “discretionary foods” are commonly used foods, not essential to cover macro and micro-nutrient needs. For most of them, it was not possible to define consumption frequencies as adequate for health. In most cases, IDGs recommended occasional consumption, limited to particular events as pleasurable, social, and tasty moments. In this group are included, among others, sweetened beverages, chips, ice creams, creamy tarts, alcoholic beverages, and processed meat. However, to overcome the problem of the acceptability of dietary plans and make them more realistic for pediatric ages some “discretionary foods” such as “sugar” and “jam” and, “cakes, spoon desserts and ice creams,” “sweet and chocolate spread” and “jam” were included with consideration for the nutrient adequacy. Items such as alcoholic beverages, fruit juices, and preserved meat were excluded from the dietary plans (Supplementary Table 1).

The database of the national food consumption INRAN-SCAI 2005–2006 survey, described in detail by Leclercq et al. (43) and Sette et al. (44) was used to obtain the nutritional composition (energy, macronutrients, micronutrients, dietary fiber) of the food subgroups used to develop the dietary plans. The INRAN-SCAI 2005–2006 Food Energy and Nutrient Composition Database was compiled considering the energy and nutrient composition of food as found in the Council for Agricultural Research and Economics–Research Centre for Food and Nutrition database (45), then completed with the Food Composition Database for Epidemiological Studies in Italy in case of missing items (46). The nutritional composition of each food subgroup was calculated as the weighted average of energy, macro, and micro-nutrients according to the consumption levels of the Italian population as resulting from the latest national survey INRAN-SCAI 2005–2006 (43). Values provided for raw foods include net waste.

### Parameters for balancing the dietary plans

In the proposed dietary plans, the daily energy (%) was divided into three main meals and two snacks (42). General acceptability was accomplished by making a trade-off between promoting healthy foods (i.e., “fruit,” “vegetables,” and “legumes”) and proposing dietary plans attractive for children and families, avoiding the monotony of food choices. Therefore, the adequacy of the dietary plans to the nutritional and energy requirements was combined with other health-promoting criteria. In summary, the following aspects were taken into consideration:

(a) The importance of including all types of foods from all food groups (including nuts);
(b) The inclusion of “legumes” as sources of vegetable proteins;
(c) The proportion between saturated and unsaturated fats;
(d) The containment of added sugars even with the selection of sweet foodstuffs;
(e) The alternate provision of pasta and soups, possibly including “legumes” and/or “vegetables” as ingredients;
(f) The presence of fresh fruit and vegetables at every meal; and
(g) The presence of red meat once a week and the avoidance of processed meat.

## Results

### Suggested portion sizes and frequencies of consumption

Tables 1–3 report the SPSs and frequencies of consumption of each food subgroup for the six classes of age. Qualitative recommendations were provided such as continuous administration of breast milk up to 2 years of age (47), the selection of lean and white meat (48), and the exclusion of large, predatory fishes that are more likely to be contaminated with metals (49).

**TABLE 1:** Suggested portion sizes (SPSs*) and consumption frequencies of food subgroups for infants aged 12–23 months and 24–47 months.

Food subgroups	12–23 months		24–47 months	
	SPSs*	Frequencies	SPSs*	Frequencies
Pasta/rice, polenta, barley, etc.	25 g	2 times/day	40 g	2 times/day
Bread	15 g	2 times/day	20 g	2 times/day
Pizza (replacing, as main course 1 SPS* of “Pasta/rice, bread etc.”)	80 g	1 time/week	80 g	1 time/week
Bakery products and breakfast cereals	1 biscuit or 1 rusk or 10 g breakfast cereals	1 time/day	2 biscuits or 2 rusks or 20 g breakfast cereals	1 time/day
Potatoes (replacing bread)	70 g	1 time/week	100 g	1 time/week
Vegetables, fresh	15 g raw (e.g., tomatoes, lettuce, etc.) or 70 g cooked (e.g., chard, spinach, etc.)	2 times/day	20 g raw (e.g., tomatoes, lettuce, etc.) or 80 g cooked (e.g., chard, spinach, etc.)	2 times/day
Fruit, fresh	40 g	3 times/day	70 g	3 times/day
Legumes	30 g or 10 g dried	3 times/week	30 g fresh or 10 g dried	3 times/week
Cow milk	150 mL	1 time/day	200 mL	1 time/day
Yogurt	60 g	1 time/day	60 g	4 times/week
Cheese	25 g fresh (e.g., mozzarella) or 15 g semi-seasoned (e.g., caciotta)	2 times/week	30 g fresh (e.g., mozzarella) or 20 g semi-seasoned (e.g., caciotta)	2 times/week
Grated cheese**	15 g seasoned (e.g., parmesan)	Over the week	30 g seasoned (e.g., parmesan)	Over the week
Fish, fresh***	30 g	3 times/week	50 g	3 times/week
Meat, fresh****	25 g red meat	1 time/week	35 g red meat	1 time/week
	25 g white meat	2 times/week	35 g white meat	2 times/week
Egg	50 g (1 egg)	2 times/week	50 g (1 egg)	2 times/week
Oil	23 g extra virgin olive oil	Over the day	30 g extra virgin olive oil	Over the day
Sweets	20 g baked desserts (e.g., tart, donut, etc.) or 10 g chocolate, jam, etc. or 40 g spoon desserts (e.g., ice cream, pudding, etc.)	1 time/week	50 g baked desserts (e.g., tart, donut, etc.) or 20 g chocolate, jam, etc. or 80 g spoon desserts (e.g., ice cream, pudding, etc.)	1 time/week
Water	1,200 mL ^AI^***** (including water from foods)	Over the day	1,200 mL ^AI^***** (including water from foods)	Over the day

*SPSs, suggested portion sizes. The quantities refer to raw food, including net waste, or, in some cases, ready for consumption (e.g., milk, bread).

**If the use of grated cheese is not appreciated, it can be replaced with a portion of cheese per week.

***Limit the consumption of large fish (e.g., tuna and swordfish).

****Prefer lean cuts and white meat (chicken, turkey, and rabbit).

*****AI, Adequate Intake.

In 12–36 months, it is recommended to continue breast milk administration whenever possible. Since it is difficult to quantify the breast milk consumed, this was not considered for nutrients coverage. Besides cow’s milk, different typologies of so-called “growth milk” are available for children 1–3 years. It is recommended to follow the advice of the pediatrician who will guide the family’s choices by evaluating the individual qualitative and quantitative needs of the infant.

**TABLE 2:** Suggested portion sizes (SPSs) and consumption frequencies of food subgroups for children aged 4–6 years and 7–10 years.

Food subgroups	4–6 years		7–10 years	
	SPSs*	Frequencies	SPSs*	Frequencies
Pasta/rice, polenta, barley, etc.	50 g	2 times/day	70 g	2 times/day
Bread	40 g	2.5 times/day	50 g	2.5 times/day
Pizza (replacing as main course 1 SPS of “Pasta/rice, bread etc.”)	150 g	1 time/week	200 g	1 time/week
Bakery products and breakfast cereals	3 biscuits or 3 rusks or 30 g breakfast cereals	1 time/day	4 biscuits or 4 rusks or 40 g breakfast cereals	1 time/day
Potatoes (replacing bread)	100 g	1 time/week	150 g	1 time/week
Vegetables, fresh	40 g raw (e.g., tomatoes, lettuce, etc.) or 120 g cooked (e.g., chard, spinach, etc.)	2 times/day	50 g raw (e.g., tomatoes, lettuce, etc.) or 150 g cooked (e.g., chard, spinach, etc.)	2 times/day
Fruit, fresh	80 g	2.5 times/day	100 g	2.5 times/day
Fruit, nuts, and seeds	20 g	3 times/week	30 g	3 times/week
Legumes	60 g fresh or 20 g dried	3 times/week	90 g fresh or 30 g dried	3 times/week
Cow milk	200 mL	1 time/day	200 mL	1 time/day
Yogurt	125 g	5 times/week	125 g	5 times/week
Cheese	40 g fresh (e.g., mozzarella) or 30 g semi-seasoned (e.g., caciotta)	2 times/week	70 g fresh (e.g., mozzarella) or 50 g semi-seasoned (e.g., caciotta)	2 times/week
Grated cheese**	20 g seasoned (e.g., parmesan)	Over the week	30 g seasoned (e.g., parmesan)	Over the week
Fish, fresh***	60 g	3 times/week	80 g	3 times/week
Meat, fresh****	45 g red meat	1 time/week	80 g red meat	1 time/week
	45 g white meat	2 times/week	80 g white meat	2 times/week
Egg	50 g (1 egg)	2 times/week	50 g (1 egg)	2 times/week
Oil	25 g extra virgin olive oil	Over the day	25 g extra virgin olive oil	Over the day
Sweets	30 g baked desserts (e.g., tart, donut, etc.) or 10 g chocolate, jam, etc. or 100 g spoon desserts (e.g., ice cream, pudding, etc.)	2 times/week	50 g baked desserts (e.g., tart, donut, etc.) or 25 g chocolate, jam, etc. or 100 g spoon desserts (e.g., ice cream, pudding, etc.)	3 times/week
Water	1,600 mL ^AI^***** (including water from foods)	Over the day	1,800 mL ^AI^***** (including water from foods)	Over the day

*SPSs, suggested portion sizes. The quantities refer to raw food, including net waste, or, in some cases, ready for consumption (e.g., milk, bread).

**If the use of grated cheese is not appreciated, it can be replaced with a portion of cheese per week.

***Limit the consumption of large fish (e.g., tuna and swordfish).

****Prefer lean cuts and white meat (chicken, turkey, and rabbit).

*****AI, Adequate Intake.

**TABLE 3:** Suggested portion sizes (SPSs) and consumption frequencies of food subgroups for adolescents aged 11–14 years and 15–17 years.

Food subgroups	11–14 years		15–17 years	
	SPSs*	Frequencies	SPSs*	Frequencies
Pasta/rice, polenta, barley, etc.	100 g	2 times/day	100 g	2 times/day
Bread	50 g	3 times/day	50 g	4 times/day
Pizza (replacing as main course 1 SPS of “Pasta/rice, bread, etc.”)	350 g	1 time/week	350 g	1 time/week
Bakery products and breakfast cereals	4 biscuits or 4 rusks or 40 g breakfast cereals	1 time/day	5 biscuits or 5 rusks or 50 g breakfast cereals	1 time/day
Potatoes (replacing bread)	200 g	2 times/week	200 g	2 times/week
Vegetables, fresh	50 g raw (e.g., tomatoes, lettuce, etc.) or 200 g cooked (e.g., chard, spinach, etc.)	2 times/day	50 g raw (e.g., tomatoes, lettuce, etc.) or 200 g cooked (e.g., chard, spinach, etc.)	2 times/day
Fruit, fresh	120 g	2.5 times/day	150 g	2.5 times/day
Fruit, nuts, and seeds	30 g	3 times/week	30 g	3 times/week
Legumes	120 g fresh or 40 g dried	3 times/week	120 g fresh or 40 g dried	3 times/week
Cow milk	200 mL	1 time/day	200 mL	1 time/day
Yogurt	125 g	1 time/day	125 g	1 time/day
Cheese	100 g fresh (e.g., mozzarella) or 80 g semi-seasoned (e.g., caciotta)	2 times/week	100 g fresh (e.g., mozzarella) or 80 g semi-seasoned (e.g., caciotta)	2 times/week
Grated cheese**	50 g seasoned (e.g., parmesan)	Over the week	50 g seasoned (e.g., parmesan)	Over the week
Fish, fresh***	150 g	3 times/week	150 g	3 times/week
Meat, fresh****	100 g red meat	1 time/week	100 g red meat	1 time/week
	100 g white meat	2 times/week	100 g white meat	2 times/week
Egg	50 g (1 egg)	2 times/week	50 g (1 egg)	2 times/week
Oil	30 g extra virgin olive oil	Over the day	40 g extra virgin olive oil	Over the day
Sweets	100 g baked desserts (e.g., tart, donut, etc.) or 40 g chocolate, jam, etc. or 125 g spoon desserts (e.g., ice cream, pudding, etc.)	4 times/week	100 g baked desserts (e.g., tart, donut, etc.) or 40 g chocolate, jam, etc. or 125 g spoon desserts (e.g., ice cream, pudding, etc.)	5 times/week
Water	2,000 mL ^AI^***** (including water from foods)	Over the day	2,250 mL ^AI^***** (including water from foods)	Over the day
Sugar******	5 g (a level teaspoon)	1 time/day	5 g (a level teaspoon)	1 time/day

*SPSs, suggested portion sizes. The quantities refer to raw food, including net waste, or, in some cases, ready for consumption (e.g., milk, bread).

**If the use of grated cheese is not appreciated, it can be replaced with a portion of cheese per week.

***Limit the consumption of large fish (e.g., tuna and swordfish).

****Prefer lean cuts and white meat (chicken, turkey, and rabbit).

*****AI, Adequate Intake.

******For sugar, a limited consumption is recommended and, in any case, not exceeding the reported amount.

As shown in Tables 1–3, items from “cereals and derivatives,” “milk,” “fruit,” and “vegetables,” “olive oil” and “water” were included daily. Pizza (1 SPS ranging from 80 g for 1–2 years infants to 350 g for 15–17 years adolescents) was included once a week, as a substitute for 1 SPS of other cereal-based products (e.g., pasta, rice, polenta, barley, etc.) as the main course. “Sweets” (e.g., baked desserts, spoon desserts, chocolate, jam, etc.) were gradually introduced, once a week in infants and up to five times a week in adolescents. To accomplish the recommendation of having a large quota of vegetable proteins (50–52), legumes were included with a frequency of three times a week, increasing the quantities in relation to the children’s age. Added sugars (1 spoon/day) were first introduced in the 11–14 age class and the amount is maintained for older children. The choice to introduce added sugars at this age was in consideration of the increased energy requirement of adolescents, permitting more flexibility in food variety, to more easily maintain the recommended quantity of sugar or the adequate intake of other foods providing intrinsic sugar (e.g., fruit and milk).

### Weekly dietary plans

The SPSs and consumption frequencies defined in Tables 1–3 were arranged in weekly dietary plans for each of the six classes of age (Tables 4–9). A disclaimer that these plans should be considered illustrative examples, not prescriptive since it relates to population groups and not to a single individual, was provided. The main critical aspect of the dietary plans was related to the monotony, especially for the snacks resulting only with the alternative of yogurt, milkshake, fresh fruit, nuts, and seeds, or bread with chocolate or tomatoes. Less problematic is the variety of the main meals (breakfast, lunch, and dinners) in which the acceptability problems could be related to the presence of legumes not frequently consumed by this age group. Given the importance the general Italian population places on the consumption of animal-source foods in the pediatric age, the SPSs could be deemed very small for the youngest (in the ages 2–6 years) for fish (50–60 g) and meat (35–45 g). This could create acceptance problems among the parents, especially in infancy and school-age children.

**TABLE 4:** Example of a weekly dietary plan for infants aged 12–23 months.

Monday	Tuesday	Wednesday	Thursday	Friday	Saturday	Sunday
**Breakfast**	**Breakfast**	**Breakfast**	**Breakfast**	**Breakfast**	**Breakfast**	**Breakfast**

Cow milk 150 mL	Yogurt 60 g	Cow milk 150 mL	Cow milk 150 mL	Cow milk 150 mL	Cow milk 150 mL	Yogurt 60 g
1 Biscuit	Breakfast cereals 10 g	1 Biscuit	1 Biscuit	Breakfast cereals 10 g	1 Rusk	1 Biscuit

**Snack**	**Snack**	**Snack**	**Snack**	**Snack**	**Snack**	**Snack**

Yogurt 60 g	Fresh fruit 40 g	Fresh fruit 40 g	Yogurt 60 g	Fresh fruit 40 g	Yogurt 60 g	Milk shake (Cow milk 150 mL and Fresh fruit 40 g)
					Fresh fruit 40 g	

**Lunch**	**Lunch**	**Lunch**	**Lunch**	**Lunch**	**Lunch**	**Lunch**

Rice 25 g	Pasta 25 g	Rice 25 g with Legumes (fresh 30 g or dried 10 g)	Pasta 25 g	Rice 25 g with Legumes (fresh 30 g or dried 10 g)	Pasta 25 g	Pasta 25 g
Fish 30 g	Red meat 25 g		Fresh cheese 25 g		Fish 30 g	1 Egg
Cooked vegetables 70 g	Raw vegetables 15 g	Cooked vegetables 70 g	Cooked vegetables 70 g	Raw vegetables 15 g	Cooked vegetables 70 g	Cooked vegetables 70 g
Bread 15 g	Bread 15 g	Bread 15 g	Bread 15 g	Bread 15 g		Potatoes 70 g
Fresh fruit 40 g	Fresh fruit 40 g	Fresh fruit 40 g	Fresh fruit 40 g	Fresh fruit 40 g	Fresh fruit 40 g	Fresh fruit 40 g

**Snack**	**Snack**	**Snack**	**Snack**	**Snack**	**Snack**	**Snack**

Fresh fruit 40 g	Milkshake (Cow milk 150 mL and Fresh fruit 40 g)	Yogurt 60 g	Fresh fruit 40 g	Yogurt 60 g	Bread 15 g with tomato	Bread 15 g with 1 teaspoon of chocolate spread

**Dinner**	**Dinner**	**Dinner**	**Dinner**	**Dinner**	**Dinner**	**Dinner**

Pasta 25 g with Legumes (fresh 30 g or dried 10 g)	Rice 25 g	Pasta 25 g	Pasta 25 g	Pasta 25 g	Pizza 80 g	Vegetable soup with Pasta 25 g
	Semi-seasoned cheese 15 g	Fish 30 g	White meat 25 g	1 Egg		
Cooked vegetables 70 g	Cooked vegetables 70 g	Raw vegetables 15 g	Cooked vegetables 70 g	Cooked vegetables 70 g		White meat 25 g
Bread 15 g	Bread 15 g	Bread 15 g	Bread 15 g	Bread 15 g		Bread 15 g
Fresh fruit 40 g	Fresh fruit 40 g	Fresh fruit 40 g	Fresh fruit 40 g	Fresh fruit 40 g	Fresh fruit 40 g	Fresh fruit 40 g

**To be added:**						
**Water** about 1,200 mL per day (including water from foods).						
23 g of **extra virgin olive oil** per day.						
15 g of **grated cheese** per week (if the use of grated cheese is not appreciated, it can be replaced with 1 portion of cheese per week).						

**TABLE 5:** Example of a weekly dietary plan for infants aged 24–47 months.

Monday	Tuesday	Wednesday	Thursday	Friday	Saturday	Sunday
**Breakfast**	**Breakfast**	**Breakfast**	**Breakfast**	**Breakfast**	**Breakfast**	**Breakfast**

Cow milk 100 mL	Cow milk 200 mL	Cow milk 100 mL	Cow milk 200 mL	Cow milk 100 mL	Cow milk 200 mL	Cow milk 100 mL
2 Biscuits	Breakfast cereals 20 g	2 Biscuits	Breakfast cereals 20 g	2 Biscuits	2 Rusks	2 Biscuits

**Snack**	**Snack**	**Snack**	**Snack**	**Snack**	**Snack**	**Snack**

Yogurt 60 g	Spoon dessert 40 g	Yogurt 60 g	Fresh fruit 70 g	Fresh fruit 70 g	Fresh fruit 70 g	Milkshake (Cow milk 100 mL and Fresh fruit 70 g)

**Lunch**	**Lunch**	**Lunch**	**Lunch**	**Lunch**	**Lunch**	**Lunch**

Rice 40 g	Pasta 40 g	Rice 40 g with Legumes (fresh 30 g or dried 10 g)	Pasta 40 g	Rice 40 g with Legumes (fresh 30 g or dried 10 g)	Pasta 40 g	Pasta 40 g
Fish 50 g	Red meat 35 g		Fresh cheese 30 g		Fish 50 g	1 Egg
Cooked vegetables 80 g	Raw vegetables 20 g	Cooked vegetables 80 g	Cooked vegetables 80 g	Raw vegetables 20 g	Cooked vegetables 80 g	Cooked vegetables 80 g
Bread 20 g	Bread 20 g	Bread 20 g	Bread 20 g	Bread 20 g		Potatoes 100 g
Fresh fruit 70 g	Fresh fruit 70 g	Fresh fruit 70 g	Fresh fruit 70 g	Fresh fruit 70 g	Fresh fruit 70 g	Fresh fruit 70 g

**Snack**	**Snack**	**Snack**	**Snack**	**Snack**	**Snack**	**Snack**

Milkshake (Cow milk 100 mL and Fresh fruit 70 g)	Yogurt 60 g	Milkshake (Cow milk 100 mL and Fresh fruit 70 g)	Yogurt 60 g	Milkshake (Cow milk 100 mL and Fresh fruit 70 g)	Bread 20 g with tomato	Bread 20 g with 1 teaspoon of chocolate spread

**Dinner**	**Dinner**	**Dinner**	**Dinner**	**Dinner**	**Dinner**	**Dinner**

Pasta 40 g with Legumes (fresh 30 g or dried 10 g)	Rice 40 g	Pasta 40 g	Pasta 40 g	Pasta 40 g	Pizza 80 g	Vegetable soup with Pasta 40 g
	Semi-seasoned cheese 20 g	Fish 50 g	White meat 35 g	1 Egg		
Cooked vegetables 80 g	Cooked vegetables 80 g	Raw vegetables 20 g	Cooked vegetables 80 g	Cooked vegetables 80 g		White meat 35 g
Bread 20 g	Bread 20 g	Bread 20 g	Bread 20 g	Bread 20 g		Bread 20 g
Fresh fruit 70 g	Fresh fruit 70 g	Fresh fruit 70 g	Fresh fruit 70 g	Fresh fruit 70 g	Fresh fruit 70 g	Fresh fruit 70 g

**To be added:**						
**Water** about 1,200 mL per day (including water from foods).						
30 g of **extra virgin olive oil** per day.						
30 g of **grated cheese** per week (if the use of grated cheese is not appreciated, it can be replaced with 1 portion of cheese per week).						

**TABLE 6:** Example of a weekly dietary plan for children aged 4–6 years.

Monday	Tuesday	Wednesday	Thursday	Friday	Saturday	Sunday
**Breakfast**	**Breakfast**	**Breakfast**	**Breakfast**	**Breakfast**	**Breakfast**	**Breakfast**

Cow milk 200 mL	Yogurt 125 g	Cow milk 200 mL	Cow milk 200 mL	Cow milk 200 mL	Cow milk 200 mL	Cow milk 200 mL
3 Biscuits	Breakfast cereals 30 g	3 Biscuits	3 Biscuits	Breakfast cereals 30 g	3 Rusks with 1 teaspoon of jam	3 Biscuits

**Snack**	**Snack**	**Snack**	**Snack**	**Snack**	**Snack**	**Snack**

Yogurt 125 g	Bread 20 g	Spoon dessert 100 g	Fresh fruit 80 g	Fresh fruit 80 g	Yogurt 125 g	Fresh fruit 80 g
Fresh fruit 40 g	Nuts and seeds 20 g				Fresh fruit 40 g	

**Lunch**	**Lunch**	**Lunch**	**Lunch**	**Lunch**	**Lunch**	**Lunch**

Rice 50 g	Pasta 50 g	Pasta 50 g with Legumes (fresh 60 g or dried 20 g)	Pasta 50 g	Rice 50 g with Legumes (fresh 60 g or dried 20 g)	Pasta 50 g	Pasta 70 g
Fish 60 g	Red meat 45 g		Fresh cheese 40 g		Fish 60 g	White meat 45 g
Cooked vegetables 120 g	Raw vegetables 40 g	Cooked vegetables 120 g	Raw vegetables 40 g	Raw vegetables 40 g	Cooked vegetables 120 g	Raw vegetables 40 g
Bread 40 g	Bread 40 g	Bread 60 g	Bread 40 g	Bread 60 g	Bread 40 g	Potatoes 100 g
Fresh fruit 80 g	Fresh fruit 40 g	Fresh fruit 80 g	Fresh fruit 80 g	Fresh fruit 40 g	Fresh fruit 80 g	Fresh fruit 80 g

**Snack**	**Snack**	**Snack**	**Snack**	**Snack**	**Snack**	**Snack**

Nuts and seeds 20 g	Milkshake (Cow milk 200 mL and Fresh fruit 80 g)	Yogurt 125 g	Fresh fruit 80 g	Yogurt 125 g	Bread 60 g with tomato	Bread 40 g with 1 teaspoon of chocolate spread
		Fresh fruit 40 g		Nuts and seeds 20 g		

**Dinner**	**Dinner**	**Dinner**	**Dinner**	**Dinner**	**Dinner**	**Dinner**

Pasta 50 g with Legumes (fresh 60 g or dried 20 g)	Rice 50 g	Pasta 50 g	Pasta 50 g	Pasta 50 g	Pizza 150 g	Vegetable soup with Pasta 30 g
	Semi-seasoned cheese 30 g	1 Egg	Fish 60 g	White meat 45 g		
Raw vegetables 40 g	Cooked vegetables 120 g	Raw vegetables 40 g	Cooked vegetables 120 g	Cooked vegetables 120 g		1 Egg
Bread 60 g	Bread 40 g	Bread 40 g	Bread 60 g	Bread 40 g		Bread 60 g
Fresh fruit 80 g	Fresh fruit 80 g	Fresh fruit 80 g	Fresh fruit 40 g	Fresh fruit 80 g	Fresh fruit 80 g	Fresh fruit 40 g

**To be added:**						
**Water** about 1,600 mL per day (including water from foods).						
25 g of **extra virgin olive oil per day**.						
20 g of **grated cheese per week** (if the use of grated cheese is not appreciated, it can be replaced with 1 portion of cheese per week).						

**TABLE 7:** Example of a weekly dietary plan for children aged 7–10 years.

Monday	Tuesday	Wednesday	Thursday	Friday	Saturday	Sunday
**Breakfast**	**Breakfast**	**Breakfast**	**Breakfast**	**Breakfast**	**Breakfast**	**Breakfast**

Cow milk 200 mL	Yogurt 125 g	Cow milk 200 mL	Cow milk 200 mL	Cow milk 200 mL	Cow milk 200 mL	Cow milk 200 mL
4 Biscuits	Breakfast cereals 40 g	4 Biscuits	4 Biscuits	Breakfast cereals 40 g	4 Rusks with 2 teaspoons of jam	4 Biscuits

**Snack**	**Snack**	**Snack**	**Snack**	**Snack**	**Snack**	**Snack**

Yogurt 125 g	Bread 25 g	Fresh fruit 100 g	Spoon dessert 100 g	Nuts and seeds 30 g	Yogurt 125 g	Fresh fruit 100 g
Fresh fruit 50 g	Nuts and seeds 30 g				Fresh fruit 50 g	

**Lunch**	**Lunch**	**Lunch**	**Lunch**	**Lunch**	**Lunch**	**Lunch**

Rice 70 g	Pasta 70 g	Pasta 70 g with Legumes (fresh 90 g or dried 30 g)	Pasta 70 g	Pasta 70 g with Legumes (fresh 90 g or dried 30 g)	Pasta 100 g	Pasta 70 g
Fish 80 g	Red meat 80 g		Fresh cheese 70 g		Fish 80 g	White meat 80 g
Cooked vegetables 150 g	Raw vegetables 50 g	Cooked vegetables 150 g	Raw vegetables 50 g	Raw vegetables 50 g	Cooked vegetables 150 g	Raw vegetables 50 g
Bread 50 g	Bread 50 g	Bread 75 g	Bread 50 g	Bread 75 g		Potatoes 150 g
Fresh fruit 100 g	Fresh fruit 50 g	Fresh fruit 100 g	Fresh fruit 100 g	Fresh fruit 100 g	Fresh fruit 100 g	Fresh fruit 100 g

**Snack**	**Snack**	**Snack**	**Snack**	**Snack**	**Snack**	**Snack**

Baked dessert 50 g	Milkshake (Cow milk 200 mL and Fresh fruit 100 g)	Yogurt 125 g	Nuts and seeds (30 g)	Yogurt 125 g	Bread 75 g with tomato	Bread 50 g with 2 teaspoons of chocolate spread
		Fresh fruit 50 g		Fresh fruit 50 g		

**Dinner**	**Dinner**	**Dinner**	**Dinner**	**Dinner**	**Dinner**	**Dinner**

Pasta 70 g with Legumes (fresh 90 g or dried 30 g)	Rice 70 g	Pasta 70 g	Pasta 100 g	Pasta 70 g	Pizza 200 g	Vegetable soup with Pasta 40 g
	Semi-seasoned cheese 50 g	1 Egg	Fish 80 g	White meat 80 g		
Raw vegetables 50 g	Cooked vegetables 150 g	Raw vegetables 50 g	Cooked vegetables 150 g	Cooked vegetables 150 g		1 Egg
Bread 75 g	Bread 50 g	Bread 50 g	Bread 75 g	Bread 50 g		Bread 75 g
Fresh fruit 100 g	Fresh fruit 100 g	Fresh fruit 100 g	Fresh fruit 100 g	Fresh fruit 100 g	Fresh fruit 100 g	Fresh fruit 50 g

**To be added:**						
**Water** about 1,800 mL per day (including water from foods).						
25 g of **extra virgin olive oil** per day.						
30 g of **grated cheese** per week (if the use of grated cheese is not appreciated, it can be replaced with 1 portion of cheese per week).						

**TABLE 8:** Example of a weekly dietary plan for adolescents aged 11–14 years.

Monday	Tuesday	Wednesday	Thursday	Friday	Saturday	Sunday
**Breakfast**	**Breakfast**	**Breakfast**	**Breakfast**	**Breakfast**	**Breakfast**	**Breakfast**

Cow milk 200 mL	Yogurt 125 g	Cow milk 200 mL	Cow milk 200 mL	Cow milk 200 mL	Cow milk 200 mL	Cow milk 200 mL
4 Biscuits	Breakfast cereals 40 g	4 Biscuit	4 Biscuits	Breakfast cereals 40 g	4 Rusks with 4 teaspoons of jam	4 Biscuits

**Snack**	**Snack**	**Snack**	**Snack**	**Snack**	**Snack**	**Snack**

Yogurt 125 g	Baked dessert 100 g	Spoon dessert 125 g	Yogurt 125 g	Fresh fruit 120 g	Yogurt 125 g	Yogurt 125 g
Fresh fruit 60 g	Nuts and seeds 30 g		Nuts and seeds 30 g		Fresh fruit 60 g	Fresh fruit 120 g

**Lunch**	**Lunch**	**Lunch**	**Lunch**	**Lunch**	**Lunch**	**Lunch**

Rice 100 g	Pasta 100 g	Pasta 100 g with Legumes (fresh 120 g or dried 40 g)	Pasta 100 g	Rice 100 g with Legumes (fresh 120 g or dried 40 g)	Pasta 100 g	Pasta 100 g
Fish 150 g	Red meat 100 g		Fresh cheese 100 g		Fish 150 g	White meat 100 g
Cooked vegetables 200 g	Raw vegetables 50 g	Cooked vegetables 200 g	Raw vegetables 50 g	Raw vegetables 50 g	Cooked vegetables 200 g	Raw vegetables 50 g
Bread 75 g	Bread 75 g	Bread 75 g	Bread 75 g	Bread 75 g		Potatoes 200 g
Fresh fruit 120 g	Fresh fruit 60 g	Fresh fruit 120 g	Fresh fruit 120 g	Fresh fruit 60 g	Fresh fruit 120 g	Fresh fruit 120 g

**Snack**	**Snack**	**Snack**	**Snack**	**Snack**	**Snack**	**Snack**

Baked dessert 100 g	Milkshake (Cow milk 200 mL and Fresh fruit 120 g)	Yogurt 125 g	Fresh fruit 120 g	Yogurt 125 g	Bread 75 g with tomato	Bread 100 g 4 with teaspoons of chocolate spread
		Fresh fruit 60 g		Nuts and seeds 30 g		

**Dinner**	**Dinner**	**Dinner**	**Dinner**	**Dinner**	**Dinner**	**Dinner**

Pasta 100 g with Legumes (fresh 120 g or dried 40 g)	Pasta 100 g	Vegetable and potatoes soup with Rice 100 g	Pasta 100 g	Pasta 100 g	Pizza 350 g	Vegetable soup with Pasta 50 g
	Semi-seasoned cheese 80 g		Fish 150 g	White meat 100 g		
Raw vegetables 50 g	Cooked vegetables 200 g	1 Egg	Cooked vegetables 200 g	Cooked vegetables 200 g		1 Egg
Bread 75 g	Bread 75 g	Bread 75 g	Bread 75 g	Bread 75 g		Bread 100 g
Fresh fruit 120 g	Fresh fruit 120 g	Fresh fruit 120 g	Fresh fruit 60 g	Fresh fruit 120 g	Fresh fruit 120 g	Fresh fruit 60 g

**To be added:**						
**Water** about 2,000 mL per day (including water from foods).						
30 g of **extra virgin olive oil** per day.						
50 g of **grated cheese** per week (if the use of grated cheese is not appreciated, it can be replaced with 1 portion of cheese per week).						
5 g of **sugar** (1 level teaspoon) per day.						

**TABLE 9:** Example of a weekly dietary plan for adolescents aged 15–17 years.

Monday	Tuesday	Wednesday	Thursday	Friday	Saturday	Sunday
**Breakfast**	**Breakfast**	**Breakfast**	**Breakfast**	**Breakfast**	**Breakfast**	**Breakfast**

Cow milk 200 mL	Yogurt 125 g	Cow milk 200 mL	Cow milk 200 mL	Cow milk 200 mL	Cow milk 200 mL	Cow milk 200 mL
5 Biscuits	Breakfast cereals 50 g	5 Biscuits	5 Biscuits	Breakfast cereals 50 g	5 Biscuits	Baked dessert 100 g

**Snack**	**Snack**	**Snack**	**Snack**	**Snack**	**Snack**	**Snack**

Yogurt 125 g	Baked dessert 50 g	Spoon dessert 125 g	Yogurt 125 g	Fresh fruit 150 g	Yogurt 125 g	Yogurt 125 g
Fresh fruit 75 g	Nuts and seeds 30 g		Nuts and seeds 30 g	Baked dessert 50 g	5 Rusks with 4 teaspoons of jam	Fresh fruit 150 g

**Lunch**	**Lunch**	**Lunch**	**Lunch**	**Lunch**	**Lunch**	**Lunch**

Rice 100 g	Pasta 100 g	Pasta 100 g with Legumes (fresh 120 g or dried 40 g)	Pasta 100 g	Rice 100 g with Legumes (fresh 120 g or dried 40 g)	Pasta 100 g	Pasta 100 g
Fish 150 g	Red meat 100 g		Fresh cheese 100 g		Fish 150 g	White meat 100 g
Cooked vegetables 200 g	Raw vegetables 50 g	Cooked vegetables 200 g	Raw vegetables 50 g	Raw vegetables 50 g	Cooked vegetables 200 g	Raw vegetables 50 g
						Potatoes 200 g
Bread 100 g	Bread 100 g	Bread 100 g	Bread 100 g	Bread 100 g		Bread 50 g
Fresh fruit 150 g	Fresh fruit 75 g	Fresh fruit 150 g	Fresh fruit 150 g	Fresh fruit 75 g	Fresh fruit 150 g	Fresh fruit 150 g

**Snack**	**Snack**	**Snack**	**Snack**	**Snack**	**Snack**	**Snack**

Baked dessert 100 g	Milkshake (Cow milk 200 mL and Fresh fruit 150 g)	Yogurt 125 g	Fresh fruit 150 g	Yogurt 125 g	Bread 100 g with tomato	Bread 100 g 4 with teaspoons of chocolate spread
		Fresh fruit 75 g		Nuts and seeds 30 g	Fresh fruit 75 g	

**Dinner**	**Dinner**	**Dinner**	**Dinner**	**Dinner**	**Dinner**	**Dinner**

Pasta 100 g with Legumes (fresh 120 g or dried 40 g)	Pasta 100 g	Vegetable and potatoes soup with Rice 100 g	Pasta 100 g	Pasta 100 g	Pizza 350 g	Vegetable soup with Pasta 50 g
	Semi-seasoned cheese 80 g		Fish 150 g	White meat 100 g		
Raw vegetables 50 g	Cooked vegetables 200 g	1 Egg	Cooked vegetables 200 g	Cooked vegetables 200 g		1 Egg
Bread 100 g	Bread 100 g	Bread 100 g	Bread 100 g	Bread 100 g		Bread 50 g
Fresh fruit 150 g	Fresh fruit 150 g	Fresh fruit 150 g	Fresh fruit 75 g	Fresh fruit 150 g	Fresh fruit 150 g	Fresh fruit 75 g

**To be added:**						
**Water** about 2,250 mL per day (including water from foods).						
40 g of **extra virgin olive oil** per day.						
50 g of **grated cheese** per week (if the use of grated cheese is not appreciated, it can be replaced with 1 portion of cheese per week).						
5 g of **sugar** (1 level teaspoon) per day.						

### Comparison of daily energy and nutrients recommendations with daily energy and nutrient intakes provided in the dietary plans

Tables 10–12 report the daily energy and nutrient content of the proposed dietary plans in the six classes of age compared to the energy and nutrient requirement according to Italian DRVs (11).

**TABLE 10:** Daily energy and nutrient intakes from the dietary plan calculated for infants (12–23 months and 24–47 months) and Italian Dietary Reference Values (DRVs)*.

	12–23 months			24–47 months		
	Italian DRVs*		Daily energy and nutrient intakes	Italian DRVs*		Daily energy and nutrient intake
	Female	Male		Female	Male	
Energy (kcal)	790	870	812	1,165	1,260	1,129
Protein (% En)	<15^(RI)^		13.7	<15^(RI)^		12.9
Total Fat (% En)	35–40^(RI)^		40.6	35–40^(RI)^		38.4
SFA (% En)	<10^(SDT)^		10.5	<10^(SDT)^		10.0
PUFA (% En)	5–10^(RI)^		8.8	5–10^(RI)^		8.4
Available carbohydrate (% En)	45–60^(RI)^		45.6	45–60^(RI)^		48.6
Total sugar (% En)	<15^(SDT)^		15.3	<15^(SDT)^		15.0
Protein (g)	14^(PRI)^		28	14^(PRI)^		37
Dietary Fiber (g)	8.4 g/1,000 kcal^(AI)^		8.8	8.4 g/1,000 kcal^(AI)^		11.1
Calcium (mg)	600^(PRI)^		526	600^(PRI)^		609
Phosphorus (mg)	460^(PRI)^		540	460^(PRI)^		688
Iron (mg)	8^(PRI)^		5	8^(PRI)^		7
Magnesium (mg)	80^(PRI)^		118	80^(PRI)^		149
Zinc (mg)	5^(PRI)^		4	5^(PRI)^		5
Vitamin A (Retinol Eq.) (μg)	300^(PRI)^		449	300^(PRI)^		496
Vitamin C (Ascorbic Acid) (mg)	35^(PRI)^		75	35^(PRI)^		90
Vitamin E (Tocopherols) (mg)	5^(AI)^		8	5^(AI)^		10
Total Folate (μg)	140^(PRI)^		180	140^(PRI)^		211
Vitamin D (μg)	15^(PRI)^		1	15^(PRI)^		1
Vitamin B_6_ (Pyridoxine) (mg)	0.5^(PRI)^		0.8	0.5^(PRI)^		1.1
Vitamin B_12_ (Cyanocobalamin) (μg)	0.9^(PRI)^		2.3	0.9^(PRI)^		3.2

*DRVs: the Dietary Reference Values for energy and macro and micro-nutrients as defined for Italy by the Levels of Reference Intake of Nutrients and Energy for the Italian Population (40). AI, Adequate Intake; PRI, population reference intake; RI, reference intake range for macronutrients; SDT, suggested dietary target; SFA, saturated fatty acids; PUFA, polyunsaturated fatty acids.

**TABLE 11:** Daily energy and nutrient intakes from the dietary plan calculated for children (4–6 years and 7–10 years) and Italian Dietary Reference Values (DRVs)*.

	4–6 years			7–10 years		
	Italian DRVs*		Daily energy and nutrient intakes	Italian DRVs*		Daily energy and nutrient intake
	Female	Male		Female	Male	
Energy (kcal)	1,433	1,553	1,467	1,818	1,980	1,872
Protein (% En)	12–18^(RI)^		14.4	12–18^(RI)^		14.9
Total Fat (% En)	20–35^(RI)^		30.5	20–35^(RI)^		28.3
SFA (% En)	<10^(SDT)^		7.6	<10^(SDT)^		7.4
PUFA (% En)	5–10^(RI)^		7.8	5–10^(RI)^		7.2
Available carbohydrate (% En)	45–60^(RI)^		55.0	45–60^(RI)^		56.7
Total sugar (% En)	<15^(SDT)^		14.6	<15^(SDT)^		14.1
Protein (g)	19^(PRI)^		53	31^(PRI)^		70
Dietary Fiber (g)	8.4 g/1,000 kcal^(AI)^		17.1	8.4 g/1,000 kcal^(AI)^		22.4
Calcium (mg)	900^(PRI)^		782	1,100^(PRI)^		925
Phosphorus (mg)	500^(PRI)^		966	875^(PRI)^		1,235
Iron (mg)	11^(PRI)^		10	13^(PRI)^		13
Magnesium (mg)	100^(PRI)^		236	150^(PRI)^		310
Zinc (mg)	6^(PRI)^		7	8^(PRI)^		10
Vitamin A (Retinol Eq.) (μg)	350^(PRI)^		605	500^(PRI)^		741
Vitamin C (Ascorbic Acid) (mg)	45^(PRI)^		110	60^(PRI)^		139
Vitamin E (Tocopherols) (mg)	6^(AI)^		10	8^(AI)^		11
Total Folate (μg)	170^(PRI)^		307	250^(PRI)^		392
Vitamin D (μg)	15^(PRI)^		1	15^(PRI)^		2
Vitamin B_6_ (Pyridoxine) (mg)	0.6^(PRI)^		1.4	0.9^(PRI)^		1.8
Vitamin B_12_ (Cyanocobalamin) (μg)	1.1^(PRI)^		3.6	1.6^(PRI)^		4.6

*DRVs: the Dietary Reference Values for energy and macro and micro-nutrients as defined for Italy by the Levels of Reference Intake of Nutrients and Energy for the Italian Population (40). AI, Adequate Intake; PRI, population reference intake; RI, reference intake range for macronutrients; SDT, suggested dietary target; SFA, saturated fatty acids; PUFA, polyunsaturated fatty acids.

**TABLE 12:** Daily energy and nutrient intakes from the dietary plan calculated for children (11–14 years and 15–17 years) and Italian Dietary Reference Values (DRVs)*.

	11–14 years			15–17 years		
	Italian DRVs*		Daily energy and nutrient intakes	Italian DRVs*		Daily energy and nutrient intake
	Female	Male		Female	Male	
Energy (kcal)	2,370	2,695	2,450	2,510	3,193	2,790
Protein (% En)	12–18^(RI)^		15.0	12–18^(RI)^		14.1
Total Fat (% En)	20–35^(RI)^		27.0	20–35^(RI)^		28.0
SFA (% En)	<10^(SDT)^		7.5	<10^(SDT)^		7.4
PUFA (% En)	5–10^(RI)^		6.5	5–10^(RI)^		6.9
Available carbohydrate (% En)	45–60^(RI)^		57.9	45–60^(RI)^		57.7
Total sugar (% En)	<15^(SDT)^		14.6	<15^(SDT)^		14.8
Protein (g)	48^(PRI)^		92	50^(PRI)^	62^(PRI)^	99
Dietary Fiber (g)	8.4 g/1,000 kcal^(AI)^		28.3	8.4 g/1,000 kcal^(AI)^		32.3
Calcium (mg)	1,300^(PRI)^		1,143	1,200^(PRI)^	1,300^(PRI)^	1,177
Phosphorus (mg)	1,250^(PRI)^		1,609	1,250^(PRI)^		1,699
Iron (mg)	10/18^(PRI)^	10^(PRI)^	16	18^(PRI)^	13^(PRI)^	18
Magnesium (mg)	240^(PRI)^		401	240^(PRI)^		430
Zinc (mg)	9^(PRI)^	12^(PRI)^	13	9^(PRI)^	12^(PRI)^	14
Vitamin A (Retinol Eq.) (μg)	600^(PRI)^		923	600^(PRI)^	700^(PRI)^	984
Vitamin C (Ascorbic Acid) (mg)	80^(PRI)^	90^(PRI)^	177	85^(PRI)^	105^(PRI)^	194
Vitamin E (Tocopherols) (mg)	11^(AI)^		13	12^(AI)^	13 ^(AI)^	16
Total Folate (μg)	350^(PRI)^		494	400^(PRI)^		538
Vitamin D (μg)	15^(PRI)^		3	15^(PRI)^		3
Vitamin B_6_ (Pyridoxine) (mg)	1.2^(PRI)^		2.5	1.3^(PRI)^		2.6
Vitamin B_12_ (Cyanocobalamin) (μg)	2.2^(PRI)^		7.0	2.4^(PRI)^		7.2

*DRVs: the Dietary Reference Values for energy and macro- and micro-nutrients as defined for Italy by the Levels of Reference Intake of Nutrients and Energy for the Italian Population (40). AI, Adequate Intake; PRI, population reference intake; RI, reference intake range for macronutrients; SDT, suggested dietary target; SFA, saturated fatty acids; PUFA, polyunsaturated fatty acids.

Energy content resulted in near the average reference values of the two sexes for each age group.

Protein content (RI) for infants is 13.7% En (12–23 months) and 12.9% En (24–47 months) in the proposed dietary plans. These values are slightly higher than those of the Italian DRVs, which recommend an RI of proteins in the range of 8–12% En for infants. For the age classes over 4 years, the protein intake falls in the recommended range of 12–18% En. When protein content is expressed as PRI (grams of proteins per day) as reported in the international recommendations (53, 54) from which the Italian DRVs were derived (40), the proposed dietary plans present some criticisms, especially in the youngest age groups with values near to the triple of the Italian DRVs (37 vs. 14.0 g/day in the 24–47 months age group, 53.0 vs. 19.0 g/day in the 4–6 years age group, and 70.0 vs. 31.0 g/day in the age group 7–10 years).

The fat content of the proposed dietary plans is in line with the Italian DRVs for all age groups. The total fat content ranging from 40.6% En in infants to 27.0% En in adolescents corresponds to the RI (35–40% En in 1–3 years and 20–35% En in the oldest groups). The total poly-unsaturated fatty acids (PUFA) content, ranging from 8.8% En in infants to 6.5% En in adolescents, corresponds to the RI of 5–10% En. Saturated fatty acid (SFA) content should be lower than 10% En (SDT) as it is the proposed dietary plan in which the content ranged from 10.5% En in infants to 7.5% En in adolescents.

The total carbohydrates content of the dietary plans, ranging from 45.6% En in infants to 57.9% En in adolescents, are in line with the RI (45–60% En) and with the indication to keep the percentage of energy around the average value of the range (50–55% En). Total sugars ranged between 15.3% En in infants and 14.6% En in adolescents, showing the difficulties of meeting the recommendations (SDT less than 15% En) in the youngest age groups.

In the proposed dietary plans, the content of dietary fiber is above the AI (8.4 g/1,000 kcal), exceeding this value from a minimum of 1.5 g/day to a maximum of 9.2 g/day for the effect of the daily quantities of fruit, vegetables, legumes, and cereals. The plant-food amount of the dietary plans was also responsible for the vitamins’ contents that resulted in line with Italian DRVs.

Calcium is the most critical nutrient of the proposed dietary plans with the recommendation (PRI) achieved, only in the 24–47 months age group (609 mg vs. 600 mg), despite the presence of milk every day, yogurt ranging from four times a week to every day, and cheese three times a week. The amount of calcium in every class of age also included the calcium supplied by drinking water. Calcium intake was 15.9% (175 mg) less than the PRI in the 7–10 years age class, 13.1% (118 mg) less than the PRI in the 4–6 years age group, 12.3% (74 mg) less than the PRI in the 12–23 months age class, and 12.1% (157 mg) less than the PRI in 11–14 years age group. The PRI for calcium was not met in the adolescents (15–17 years) accounting for 1,177 mg as both sexes’ average vs. the PRI of 1,200 mg for females and 1,300 mg for males. An increase of SPSs and/or frequencies of consumption of calcium source foods (e.g., milk, yogurt, and cheese) that would compensate for the limited amount of calcium in the dietary plans was not feasible since this would create a further imbalance of protein and fat contents. In addition to that, a larger amount of milk and dairy products would impact fat and sugars which would become too high, exceeding the recommended range.

Iron is another critical nutrient. The intake of iron meets recommendations for children over 7 years, while in the youngest groups, it was lower than recommended (PRI), 3 mg less than the PRI in the 12–23 months group, 1 mg less than the PRI in the 24–47 months group, and the 4–6 years group. Iron source foods (e.g., meat, legumes) could not be increased to avoid a further imbalance of protein intake.

## Discussion

This work demonstrated that it is possible to convert the evidence from nutrient-based research, such as recommendations on sugars, fats, fruit and vegetable, red meat, and inclusion of all foods from all food groups, into dietary guidelines as stated in the first research question of the paper. However, obstacles and critical issues were highlighted with particular regard to recommendations related to proteins, calcium, and iron. These criticisms appear particularly evident when the dietary plans are proposed every week and for all meals with respect to the provision of plans that include only lunches for one or more weeks as happens in the menus for schools or canteens. The limits of the translation of the Italian DRVs in dietary plans for pediatric age, as presented in this paper, must be taken into consideration in light of the difficulties encountered in their preparation that could be exacerbated when the plans are implemented.

The congruence between protein recommendations and the real practical possibility of developing a dietary plan is a key aspect to consider. According to our data, the quantity of protein in grams per day (PRI) was consistently higher than the DRVs, especially in the youngest age classes. However, it should be remembered that the PRI for proteins corresponds to the minimum amount of proteins sufficient to ensure nitrogen balance and that the PRI does not necessarily correspond to the optimal intake (55). For adults and school-age children, the EFSA (54) reported that protein intake doubles the recommended quantity expressed as PRI could be considered acceptable, especially considering that these quantities of proteins are frequently observed in industrialized countries. In our dietary plans, the amount of proteins (in grams per day) largely exceeded twice the PRI in infants. The question at this point is whether it is appropriate to use grams per day as a reference for the preparation of dietary plans since it is almost impossible to comply with the PRI.

According to the Italian DRVs, for children younger than 2 years, protein RI should be in the range of 8–12% En. However, as demonstrated in our study, in practice, this RI of protein is difficult to achieve and poses problems in relation to other nutrient intakes, in particular iron and calcium. The reasons for this very restrictive approach toward protein intake in infancy were related to the data obtained by Koletzco et al. (56), reporting that the administration of infant formula with high protein concentration was positively associated with increased risks of overweight and obesity, continuing beyond the first 2 years of life. In the dietary plans presented in this paper, for infants above 12 months of age, infant formula was not included following the recommendations of the European Society for Pediatric Gastroenterology, Hepatology and Nutrition (ESPGHAN) (57) that introduced cow’s milk after the first year of life. The philosophy to the approach of the present dietary plans is to include foods of common use to ensure the availability and affordability of healthy food choices (58). In addition to that, recent data support the idea that such very restrictive protein content as fixed in Italian DRVs seems to be unjustified. In fact, according to several studies (59–62), the quantity of protein that becomes problematic is above 15%. For these reasons, the ESPGHAN current recommendation (63) is to limit protein intake to 15% of total energy for infants and toddlers, without a disclaimer of keeping the values necessarily lower. This recommendation was endorsed by the consensus position statement on the diagnosis, treatment, and prevention of pediatric obesity of the Italian Society of Pediatric Endocrinology and Diabetology and the Italian Society of Pediatrics, which recommended a protein intake of less than 15% of the daily energy intake in the first 2 years of life (32, 64). Interestingly, the level of evidence and grade of this recommendation is classified as B-II (65) meaning with evidence from randomized controlled studies and/or systematic reviews of randomized studies (Evidence B), although with limits on the robustness of the recommendation (Level II). These limits could be related to the fact that the impact of protein on infant growth and overweight needs to be evaluated from both the quantity and quality perspectives. Although most studies demonstrated a positive association between protein quantity and weight gain, the differential effects of protein from various common sources, such as meat, dairy, and plants, are still unclear. Current research suggests that dairy protein has a strong effect on growth and the risk of being overweight, but effects from meat are still unclear, and the findings are discordant. Emerging research showed that meat may not have the same weight-accelerating effect as dairy protein does (62). In this sense, the proposed dietary plans included, from an early age, alternate protein sources, either animal: meat (three times a week), fish (three times a week), eggs (two times a week), or vegetables: legumes (three times a week), thus are in line with the idea of balancing not only the quantity but also the quality of the proteins to be included in dietary plans for pediatric age. An appropriate quantity of vegetal proteins (legumes) would have positive nutritional impacts in terms of fiber and micronutrient intake. Knowledge of effective alternatives for meat, fish, and dairy proteins would also have educative effects promoting the awareness of the importance of food choices, as well as the importance of more sustainable options. In fact, as pointed out by Mazzocchi et al. (66), the complementary feeding period is recognized to be a critical window for the promotion of optimal growth and of healthy behavioral development. Many factors influence the future nutritional habits of the infant and, according to this perspective, it is important to imprint a dietary approach based also on sustainable values. Thus, the early introduction of foods normally not consumed in childhood and adolescent ages, such as legumes, into the diets of infants would improve their acceptance in the future of children’s development.

Other critical aspects of current Italian DRVs to take into consideration in the preparation of dietary plans for pediatric ages are related to the energy requirements based on the reference weight obtained by Cacciari et al. (67) and calculated considering the median value of the PALs as outlined by the Scientific Advisory Committee on Nutrition (SACN) report on the Italian DRVs for the energy of the United Kingdom (68). The use of reference weight as reported by Cacciari et al. (67) has the advantage of being based on an Italian cohort. However, the consensual recommendation of the Italian Pediatricians (64) is to use the reference weight for developmental age as defined by WHO (69). The recommendation of using the WHO standard is based on the need to propose a reference system that, although not the ideal model, has greater sensitivity in identifying children and adolescents with overweight and obesity which could be particularly appropriate considering the pediatric obesity epidemic in Italy (64). On the contrary, the use of the Italian cohort (67) underestimated the prevalence of obesity compared to WHO, probably because they were based on measurements taken during the epidemic increase of obesity (70). PALs derived from the SACN (68) mainly included studies from northern European countries and, since PAL values vary considerably according to lifestyle, geographic habitat, and socioeconomic conditions (71), the application of these values to Italian children could result in an overestimation of the quota of energy requirement related to physical activity. However, examples of populations with PALs in the lower range, or less active than average, are due to children and adolescents who spend several hours every day at school or in sedentary occupations, do not practice physical sports regularly, generally use motor vehicles for transportation, and spend most leisure time on indoor activities that require little physical effort, such as watching television, reading, using computers or playing without much body displacement (72). These conditions correspond to the general sedentary attitude of Italian children and adolescents. As reported in the school-age children surveillance data of 2019 (73), 44.5% of Italian children spent more than 2 h a day in screen-watching activities and 73.6% did not walk or use bike to go to the school. Adolescents showed similar behaviors in terms of sedentary habits; according to the surveillance data related to the class of age 11–15 years (74), less than 10% of adolescents perform at least 1 h a day of physical activity, and one-fourth of them spent more than 2 h per day in screen time activities. It is worthwhile to mention that the overestimation of energy requirements using current Italian DRVs globally affects the general balance of other nutrient provisions including the RI of proteins.

Calcium and iron are critical micronutrients in our dietary plans. The coverage for calcium in the food plans was achieved only in the age group 24–47 months, despite the administration of milk every day, yogurt from five times a week to every day, and cheese three times a week. The calculation also considers the calcium content of the water as reported by Sette et al. (44). The coverage of the iron requirement in the dietary plans is achieved from 7 years onward, while in the first three classes of age, it is lower than the Italian DRVs, despite the presence of iron-containing foods (e.g., meat, legumes, etc.). Iron intake adequacy needs to be strongly monitored particularly during infancy and, for females, during adolescence. At the time of weaning, additional recommendations should be provided to include foods containing iron with high bioavailability and substances that favor its absorption, such as ascorbic acids and amino acids; it is worth mentioning that meat and fish not only contain heme iron, with high bioavailability but also favor the absorption of non-heme iron in the same meal (75). The limitation of the protein content affected the iron and calcium quantity of the dietary plans. The very restrictive recommendations on proteins for children younger than 2 years as reported in the present version of the DRVs (8–12% En) should also be reconsidered for iron and calcium coverage.

According to Italian DRVs, calcium requirements for developmental ages ranged from 600 mg/day in infants to 1,300 mg/day in adolescents. These quantities are difficult to achieve with a diet that does not include fortified foods and/or supplements even increasing the protein quantity. According to the EFSA (76), the recommendations for calcium in this age group ranged from 450 mg/day to 1,150 mg/day meaning from 25 to 12% less than the Italian DRVs. Calcium EFSA recommendations were recently adopted by other European countries, such as France (77) and Spain (78). A rethinking should also be carried out for Italian DRVs in relation to these differing values.

The proposed dietary plans have a large quota of vegetable products hence the vitamins and fiber quantities that are in line with Italian DRVs. The quantity of plant foods, together with the provision of white meat and extra-virgin olive oil is responsible for the adequate fat intake as well as the containment of SFAs in favor of monounsaturated fatty acids. The dietary plans presented in this work represent an important element of congruence between healthy nutritional choices and sustainability, as claimed by the EAT-Lancet Commission on healthy diets and sustainable food systems (58).

Current Italian children’s food consumption pattern strongly differs from the proposed dietary plans (73). Breakfast and snacking habits, fruit, vegetable, and legume consumption, are far from the recommendations for pediatric ages. The habit of skipping breakfast (8.7%) or consuming it inadequately (35.6%) would persist when children grow up. Above half of the Italian children (55.2%) reported mid-morning snack consumption as more excessive than recommended. One-fourth (24.3%) consumed fruit and vegetable less than once a day. Daily intake of sugary and/or carbonated drinks was reported by 25.4%. Legumes are consumed less than once a week by 38.4% of children. The consumption of sweets and savory snacks was reported more than three times a week by 48.3 and 9.4%, respectively. A similar situation is reported in Italian adolescents (74) in which the habit of skipping breakfast was reported by 25.5%. Only one-third of adolescents consume fruit and vegetables at least once a day and 28% did not consume legumes or consume them less than once a week. Consumption of sugary/carbonated drinks is commonly reported (43% from 1 to 4 times a week).

It is important to realize that there is a gap between current dietary practices and recommended diets for infants, children, and adolescents. Sufficient population-based data exist to identify the magnitude of the problem and design targeted interventions. Areas to consider include the appropriateness of total caloric intake, eating patterns, the balance of foods/beverages chosen from each food group, and the intake of specific nutrients (79). These considerations led to the main limitations of the proposed dietary plans which are related to their acceptability. Even without conducting an intervention study, we could speculate that the level of acceptability of the proposed dietary plans would be limited, considering that they are quite far from the current dietary habits. Compliance with the DRVs severely limits the acceptability and pleasantness of dietary plans with breakfasts and snacks that appear monotonous and repetitive. The issue of monotony is not only related to the acceptability; diversity of the diet and variety of foods offered to children are important to avoid selective food choices attitudes and neophobia (80). During the third year of life, most children enter a neophobic phase during which previously liked foods are no longer accepted and the introduction of new foods becomes difficult. For these reasons, it is crucial to promote the habits of eating a variety of foods before the neophobic phase since this can continue further on into childhood, adolescence, and adulthood (81). In other terms, exposure to more varied foods during the early stage of development is associated with more varied diets through young life and into adulthood when variety is one of the requirements of a healthy diet (82). Furthermore, for some age classes, the proposed SPSs could be deemed very small, limiting the acceptance of recommendations by parents. In addition to that, the strong restriction of sweet products following the recommendations limit of added sugars (24, 83, 84) exacerbated the monotony of food choices, especially for breakfast and snacks that in some cases could appear as difficult application and even with the inclusion of “discretionary foods” for children more than that was recommended for adults (24).

This paper has strengths and limitations. The possibility of using the national database in which the combination of foods was weighted according to the consumption levels of the Italian population as resulting from the latest national survey INRAN-SCAI 2005–2006 (43), is an important added value to the methodology that guaranteed the results more accurately represented the realities of those who would utilize them. No other commercial database has this power. The present dietary plans were conceived as a key and new element of the Italian Guidelines for healthy eating (25), meaning dietary plans addressed to the general public and having the purpose of providing parents with advice on how to organize meals for children. The limitation of the paper and the limitation of the approach are related to the fact that in transferring this information to the consumers it is important to emphasize the illustrative and non-prescriptive nature of the proposed dietary plans, which are related to the total population and would not replace the counseling of the pediatrician as the first reference for parents. However, the proposed SPSs and frequencies of consumption can be a useful tool in community dietary planning and could be the reference for determining the approach to designing menus for infants, children, and adolescents.

### Conclusion

We demonstrated that the translation of nutrient recommendations, based on the best available scientific evidence into nutritionally adequate dietary plans for pediatric ages is a complex exercise both for nutrient adequacy and acceptance of the dietary plans by children and by extension their parents. The results of this study can be of great interest to different stakeholders in school-feeding activities, as well as to some other food service areas such as company service canteens, chain restaurants, or other individual establishments. The dietary plans are conceived to be adequate with regard to food composition, caloric and nutrient intakes, and food variety. However, their implementations require action, information, and advocacy, for which the pediatricians could have a very important role. In fact, the acceptability of the proposed dietary plans is an open question that should be addressed in further studies measuring their satisfaction, liking, and acceptance.

The strength of scientific evidence for developing nutrient recommendations often becomes weak when translating into daytime actions, mainly because the diet is intrinsic to culture and behavior more than the calories and the nutritional content of foods. In this sense, the development of guidelines for healthy eating is a task that is more than a communication exercise of writing practical recommendations, instead, it is a scientific process unexempt from criticisms. Potential criticisms could be limited if appropriately supported by evidence and pursued with a consensual approach. Finally, the main message that we want to transmit with this paper is that the development of the Italian DRVs should consider the equilibrium of the overall daily food intake, consider the nutrient recommendations in the framework of the diet and that the large part of nutritional recommendations must be achievable with real foods and not with the necessity of supplements or fortified foods (58, 82).

## Data availability statement

The original contributions presented in the study are included in the article/Supplementary material, further inquiries can be directed to the corresponding author.

## Members of the Working Group on Pediatric Nutrition of Italian Dietary Guidelines

Margherita Caroli, Marcello Giovannini, Giuseppe Morino, Silvia Scaglioni, Andrea Vania, and Elvira Verduci.

## Author contributions

LR, RP, DM, and AG were carried out the research questions, conceptualization, and design of the study. RP and DM were revised the methodology. RP, DM, and PB were carried out the database compilation. LR was carried out the manuscript writing and original draft preparation. RP, DM, PB, and AG were done writing, review, and editing. AG is the President and LR is the General Coordinator of the Commission of Revision of IDGs. All authors and the members of the Working Group on Pediatric Nutrition of Italian Dietary Guidelines have read and agreed to the published version of the manuscript.
