# Supplementary material for: Considerations for the translation of nutrient recommendations as dietary plans for infants, children, and adolescents as reported in Italian Guidelines for healthy eating
Source: Front Nutr. 2022 Aug 25;9:935963. doi: 10.3389/fnut.2022.935963 (PMC9452650; doi:10.3389/fnut.2022.935963)
Supplement: Supplementary file 1 [file Table_1.DOCX]

Supplementary Material

**Table 1 - List of food main groups, groups and subgroups of the Italian Dietary Guidelines and the food subgroups considered in dietary plans for pediatric age.**

| **FOOD GROUPING OF ITALIAN DIETARY GUIDELINES (ADULT)** | | | **DIETARY PLANS FOR PEDIATRIC AGE** |
| --- | --- | --- | --- |
| **MAIN GROUPS** | **GROUPS** | **SUBGROUPS** | **SUBGROUPS SELECTION** |
| **CEREALS AND DERIVATIVES, TUBERS** | CEREALS AND DERIVATIVES | bread | X |
|  |  | bread substitutes |  |
|  |  | pasta, rice, corn, spelled, barley, etc. | X |
|  |  |  | pizza* |
|  |  | bakery products | X |
|  |  | breakfast cereals | X |
|  | TUBERS | potatoes | X |
| **FRUIT AND VEGETABLES** | FRUIT | fruit, fresh | X |
|  |  | dried/dehydrated unsweetened fruit |  |
|  | VEGETABLES | vegetables, fresh | cooked vegetables** |
|  |  | leafy salad | raw vegetables** |
| **MEAT, FISH, EGGS, LEGUMES** | MEAT | red meat | X |
|  |  | white meat | X |
|  | FISH | fish, fresh or frozen | X |
|  |  | fish, preserved |  |
|  | EGGS | eggs | X |
|  | LEGUMES | legumes, fresh | X |
|  |  | legumes, dried | X |
| **MILK AND DERIVATIVES** | MILK AND DERIVATIVES | milk | X |
|  |  | yoghurt/fermented milk | yogurt**** |
|  |  | low-fat cheese <25% fat |  |
|  |  | fatty cheeses >25% fat |  |
|  |  |  | soft cheese*** |
|  |  |  | semi-seasoned cheese*** |
|  |  |  | seasoned cheese*** |
| **SEASONING FATS** | OILS AND FATS | olive oil | extra virgin olive oil**** |
|  |  | other vegetables oil |  |
|  |  | butter |  |
|  |  | other animal fats |  |
|  |  | vegetables fats |  |
| **NUTS AND SEEDS** | NUTS AND SEEDS | nuts and seeds | X |
| **WATER** | WATER | water | X |
| **DISCRETIONARY** | SWEETS AND SNACKS | cakes, spoon desserts, ice cream | X |
|  |  | snacks, chips, bars, chocolate | chocolate**** |
|  |  | sweet and chocolate spreads | X |
|  |  | dried/dehydrated sweetened fruit |  |
|  | NERVINE BEVERAGES AND HERBAL TEAS NOT PACKAGED | tea and herbal teas |  |
|  |  | coffee |  |
|  | NON-ALCOHOLIC BEVERAGES |  |  |
|  |  | fruit juices, iced tea, soft drinks |  |
|  | ALCOHOLIC BEVERAGES | wine |  |
|  |  | beer |  |
|  |  | vermouth / aperitifs |  |
|  |  | hard alcohol |  |
|  | PROCESSED AND PRESERVED MEATS | processed and preserved meats cooked and raw ham, bresaola, speck, bacon, mortadella, salami, canned meat, etc. |  |
|  |  |  |  |
|  | SUGAR, HONEY AND JAM | sugar | X |
|  |  | honey and jam | jam**** |

***** Pizza was included in the food plans to replace 1 Suggested Portion Size (SPS) of "Pasta / rice, polenta, barley, spelled, etc."

****** Indicated with different name in the Italian Guidelines

******* The present nomenclature resulted most comprehensible

**** Of these food subgroups only the food typology indicated was included in the dietary plans
